# The Cancer Hub Approach for Upper Gastrointestinal Surgery During COVID-19 Pandemic: Outcomes from a UK Cancer Centre

**DOI:** 10.1245/s10434-022-12571-4

**Published:** 2022-10-18

**Authors:** Joseph P. Doyle, Pranav H. Patel, Sophie L. F. Doran, Long R. Jiao, David Cunningham, David Nicol, Vasileios K. Mavroeidis, William H. Allum, Asif M. Chaudry, Ricky H. Bhogal, Sacheen Kumar

**Affiliations:** 1 Department of Surgery, The Royal Marsden Hospital, London, UK; 2 GI Unit, The Royal Marsden Hospital, London, UK; 3 Upper GI Surgical Oncology Research Group, The Institute for Cancer Research, London, UK; 4 Digestive Disease & Surgery Institute, Cleveland Clinic London Hospital, London, UK

## Abstract

**Background:**

The coronavirus disease 2019 (COVID-19) pandemic caused unprecedented disruption to global healthcare delivery. In England, the majority of elective surgery was postponed or cancelled to increase intensive care capacity. Our unit instituted the ‘RM Partners Cancer Hub’ at the Royal Marsden Hospital in London, to deliver ongoing cancer surgery in a ‘COVID-lite’ setting. This article describes the operational set-up and outcomes for upper gastrointestinal (UGI) cancer resections performed during this period.

**Methods:**

From April 2020 to April 2021, the Royal Marsden Hospital formed the RM Partners Cancer Hub. This approach was designed to coordinate resources and provide as much oncological treatment as feasible for patients across the RM Partners West London Cancer Alliance. A UGI surgical case prioritisation strategy, along with strict infection control pathways and pre-operative screening protocols, was adopted.

**Results:**

A total of 231 patients underwent surgery for confirmed or suspected UGI cancer during the RM Partners Cancer Hub, with 213 completed resections and combined 90-day mortality rate of 3.5%. Good short-term survival outcomes were demonstrated with 2-year disease free survival (DFS) and overall survival (OS) for oesophageal (70.8% and 72.9%), gastric (66.7% and 83.3%) and pancreatic cancer resections (68.0% and 88.0%). One patient who developed perioperative COVID-19 during the RM Partners Cancer Hub operation made a full recovery with no lasting clinical sequelae.

**Conclusion:**

Our experience demonstrates that the RM Partners Cancer Hub approach is a safe strategy for continuing upper gastrointestinal (GI) resectional surgery during future periods of healthcare service disruption

Severe acute respiratory syndrome coronavirus 2 (SARS-CoV-2) was first reported in December 2019 in China, and by March 2020 coronavirus disease 2019 (COVID-19) was declared a pandemic by the World Health Organization. Initially, the potential impact of the pandemic upon healthcare systems and the delivery of cancer surgery remained unknown. Early in the global response, healthcare resources were re-allocated to treat the unprecedented numbers of patients with COVID-19, particularly in the critical care setting. In England, the need to increase intensive care capacity resulted in the majority of healthcare trusts cancelling or postponing elective surgery, which in many instances involved cancelling cancer surgery.^1–4^ Whilst these measures were necessary for the National Health Service (NHS) to manage the immediate and rising numbers of COVID-19 admissions, there was a definite risk of disease progression and inferior outcomes in patients with malignancy—none more so than for patients with upper gastrointestinal (UGI) cancers [henceforth UGI cancer pertaining to both oesophagogastric (OG) and hepatopancreatobiliary (HPB) cases].^1,3^ To deliver cancer surgery during the pandemic, new patient pathways had to be rapidly instituted, and the delivery of these became the priority in the field of surgical oncology.^2,3,5^ The challenge facing clinicians was two-fold: establishing safe efficient pathways that enabled patients to undergo oncological UGI resections and minimising COVID-19 transmission during the peri-operative period.^1,5,6^ This is underscored by our initial report of three patients developing COVID-19 pneumonia having undergone HPB interventions at the beginning of the pandemic.^7^ The risk of patients contracting perioperative COVID-19 while undergoing major surgery was of primary concern, our understanding of which evolved as the pandemic progressed; a study by the COVIDsurg collaborative published in May 2020 showed that pre-operative COVID-19 infection was associated with a 23.8% 30-day operative mortality.^8^ Using these initial clinical experiences, our unit engaged in the ‘RM Partners Cancer Hub’ structure based at The Royal Marsden Hospital (RMH) in London to allow cancer surgery to continue in a ‘COVID-19-lite’ setting.^9^ We present the operational set-up of the Hub structure and the outcomes of patients undergoing UGI cancer resections during this period.

## Methods

### Data Collection and Analysis

The data collected for all patients included in the RM Partners Cancer Hub was curated in a prospectively updated database. All patients were consented for the surgical procedure and informed of the specific risks of peri-operative COVID-19 infection. All data were managed according to the Caldicott principles; these are fundamental principles that NHS organisations are required to follow to protect any information that could identify a patient, which ensure that patient information is used and shared only when it is appropriate to do so.

The results are expressed as median and range. Statistical analysis was conducted using SPSS version 24.0 (Statistical Package for the Social Sciences, IBM, USA). Disease-free survival (DFS) and overall survival (OS) were evaluated by the Kaplan–Meier (KM) method, with the latter KM survival curves generated using GraphPad (GraphPad software, San Diego, USA). Survival data are presented up to 24 months from operative intervention, with right censoring adopted for those patients alive without meeting this follow-up threshold.

### Cancer Hub Structure and Remit

In April 2020, a new strategy to commission cancer care was adopted regionally across London, and The Royal Marsden Hospital (RMH) became the designated RM Partners Cancer Hub. This approach was designed to coordinate services and resources to ensure the ongoing delivery of as much oncological treatment and cancer surgery as feasible by allowing NHS-independent hospitals to partner and provide time-critical treatment at non-acute hubs. This structure allowed patients deemed appropriate for oncological resection at local centres to be referred to the Cancer Hub and have their surgery prioritised on the basis of the Cancer Hub guidelines.^9^ This strategy was developed to provide continued access to cancer surgery for all patients with UGI cancer across the RM Partners West London Cancer Alliance. Every patient referred was reviewed at a specialist UGI Multidisciplinary (MDT) Meeting, with personalised treatment plans formulated taking into account tumour biology, alternative treatment (e.g. extended chemo/chemoradiotherapy) and peri-operative risk and potential resource implications.^9^ Surgery was performed under the remit of the RM Partners Cancer Hub with the principal site for UGI surgery being The Royal Marsden Hospital (RMH), London. The RM Partners Cancer Hub structure was in place from April 2020 to April 2021 inclusive before a return to the pre-pandemic patient pathway.

### Infection Control Policy

**Figure 1:**
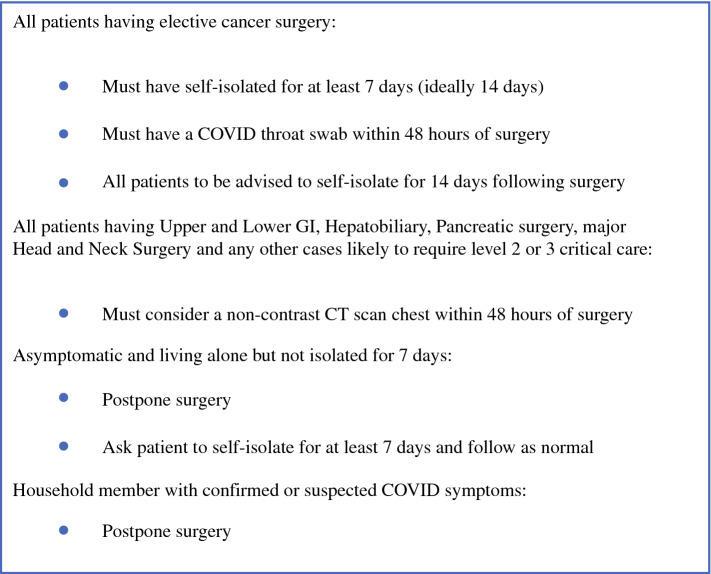
RM Partners Cancer Hub peri-operative COVID-19 screening protocol

Pre-pandemic, RMH operated as an elective centre for surgical oncology treatment, and hence, it was structurally feasible to convert the site to a ‘clean site’ for cancer surgery.^5^ Given that surgery was taking place throughout the pandemic, patient pathways were divided into low, medium and high risk. Low-risk patients were defined as asymptomatic, with no COVID-19 contact and confirmed negative SARS-CoV-2 test within 72 h and compliance with isolation guidance. Medium risk included asymptomatic patients with no COVID-19 contacts who had not fulfilled isolation criteria. High risk included cases of confirmed or suspected COVID-19, close contacts with positive cases or those who had travelled through red list countries in the preceding 14 days. The Critical Care Unit (CCU) was separated into corresponding zones to minimise the risk of nosocomial infection of post-operative patients. All patients scheduled for surgery were required to self-isolate for at least 7 days and undertake a telephone COVID-19 screening questionnaire and a pre-operative COVID-19 polymerase chain reaction (PCR) test no more than 48 h before planned surgery. In addition, a non-contrast computed tomography (CT) scan of the thorax was performed for all those patients requiring level 2 or 3 post-operative care in the early phase of the pandemic^10^ (Fig. 1). Specifically, telephone pre-assessment of patients was conducted by an experienced pre-assessment nurse, and pre-operative blood tests, electrocardiograms, specific medication instructions or other relevant investigations were performed at the time of PCR testing. Regular asymptomatic testing was offered to all hospital staff, and the mandatory use of personal protective equipment including filtering face piece respirator class 3 (FFP-3) masks, eye protection, gloves and fluid-resistant gowns was implemented for all theatre personnel present within 21 min of intubation or other aerosol generating procedure. Protocols for ‘donning’ and ‘doffing’ were followed and surface decontamination performed after every case.

### Upper Gastrointestinal Case Prioritisation

**Figure 2:**
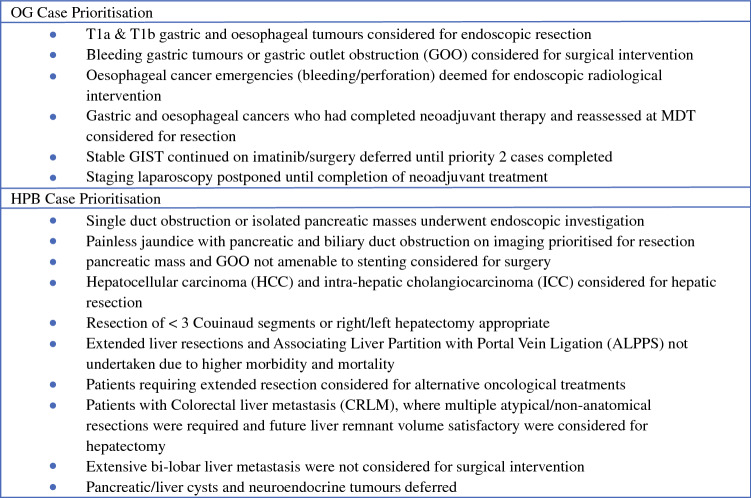
UGI Case prioritisation schedule

During the RM Partners Cancer Hub period, a clinical prioritisation schedule for all upper GI cancer surgery was implemented in accordance with the Association of Upper Gastrointestinal Surgeons (AUGIS) recommendations (Fig. 2).^9^ Case prioritisation was determined by performance status and tumour biology. Referrals were categorised as per the NHS England clinical guide for management of patients with cancer during the coronavirus pandemic^11^ into the corresponding levels:

- Priority level 1a: emergency operation needed within 24 h to save life
- Priority level 1b: urgent operation needed within 72 h
- Priority level 2: elective surgery with the expectation of cure (within 4 weeks to save life/progression of disease beyond operability)
- Priority level 3: elective surgery that can be delayed for 10–12 weeks and will have no predicted negative outcome
- Priority level 4: all other elective surgery

Oesophageal cancer emergencies were managed with endoscopic or radiological interventions, owing to their associated poor prognosis and the need for prolonged critical care stay following surgical intervention. Patients presenting with gastric outlet obstruction (GOO) or bleeding gastric tumours were considered for surgical management. T1a and T1b oesophageal and gastric tumours were initially considered for endoscopic resection. However, given the associated risk of aerosol generated associated with endoscopy, this approach was not instituted and patients were considered for definitive surgery; patients with T2 or higher stage cancers who had completed neo-adjuvant therapy and were deemed resectable at MDT restaging were considered for definitive surgery.^9^ For pancreatic cancer, endoscopy services were rationalised to investigate single duct obstruction or pancreatic masses; those patients with both pancreatic and biliary duct obstruction with associated mass were prioritised for surgery. Liver resection of 3 Couinaud segments or hemi-hepatectomy was deemed appropriate during the RM Partners Cancer Hubs operation—extended liver resections and associating liver partition with portal vein ligation (ALPPS) were not performed owing to higher associated morbidity and mortality, and those requiring extended resection were considered for alternative systemic therapy.^9^ Those with colorectal liver metastases amenable to radiofrequency/thermal ablation were considered for this with curative or bridging intent. Stable gastrointestinal stromal tumours (GISTs) were to continue on imatinib and surgery deferred until all priority cases were performed. Surgery for pancreatic cysts, liver cysts and neuroendocrine tumours were similarly deferred.

## Results

**Figure 3:**
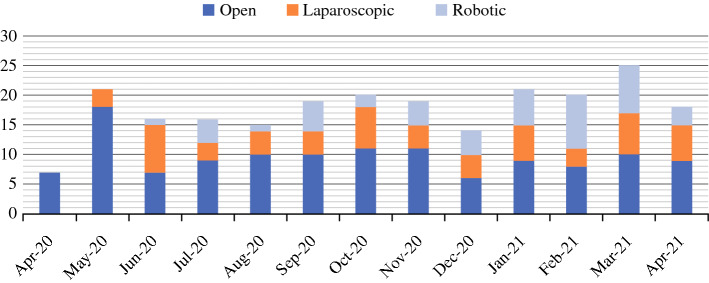
Primary operative approach for UGI cases by month; RM Partners Cancer Hub

In total, 231 patients underwent surgery for UGI cancers during the study period, with 213 completed resections (Table 1). Initially, the majority of these cases were performed via an open approach, in accordance with early advice from the Royal Colleges of Surgeons regarding the use of laparoscopy and risk of aerosolised virus transmission.^12^ However, with the publication of further evidence as well as the adoption of closed-circuit laparoscopic insufflation technologies, minimally invasive approaches were reinstituted. The breakdown of the surgical approaches are as follows: open procedures (*n* = 125, 54.1%), laparoscopic procedures (*n* = 59, 25.5%) and robotic procedures (*n* = 47, 20.3%) (Fig. 3). The breakdown of case priority levels is presented in Table 1. Post-operative CCU stay, hospital stay and 90-day mortality rates by surgical procedure are as displayed in Table 2, with combined 90-day mortality rate of 3.5%. The complication rate for all surgical procedures performed, as per the Clavien–Dindo classification, is outlined in Table 3.

**Table 1:** Case priority level breakdown

RM Partners Cancer Hub referral category	Number (%)
Priority level 1	2 (0.9%)
Priority level 2	225 (97.4%)
Priority level 3	1 (0.4%)
Priority level 4	1 (0.4%)
Unspecified	2 (0.9%)

**Table 2:** UGI procedural frequency, CCU length of stay and hospital length of stay for duration of the RM Partners Cancer Hub

Procedure	Number of cases	Primary surgical approach—*n* (%)			CCU stay (days)—median (range)	Length of stay (days)—median (range)	90-Day mortality—*n* (%)
		Open	Laparoscopic	Robotic			
*OG procedure*							
Ivor–Lewis oesophagectomy	45	3 (6.7%)	39 (86.7%)	3 (6.7%)	4 (2–77)	12 (8–85)	2 (4.4%)
Total gastrectomy	22	19 (86.4%)	1 (4.5%)	2 (9%)	1 (1–8)	11 (7–25)	0 (0%)
Subtotal gastrectomy	2	1 (50%)	0 (0%)	1 (50%)	1 (1–1)	8.5 (8–9)	0 (0%)
Left thoraco-abdominal oesophago-gastrectomy	2	1 (50%)	0 (0%)	1 (50%)	8 (2–14)	17.5 (13–22)	0 (0%)
Resection of gastric/small bowel GIST	15	4 (26.7%)	8 (53.3%)	3 (20%)	1 (0–2)	6 (3–13)	0 (0%)
Unresectable gastric-gastrojejunostomy bypass/other	4	1 (25%)	3 (75%)	0 (0%)	1 (0–1)	14 (6–21)	1 (25%)
Distal oesophagectomy + right lower lobectomy	1	1 (100%)	0 (0%)	0 (0%)	20	31	0 (0%)
Excision of complex mediastinal mass	1	1 (100%)	0 (0%)	0 (0%)	2	8	0 (0%)
Excision of diaphragmatic mass	1	1 (100%)	0 (0%)	0 (0%)	0	2	0 (0%)
*HPB procedure*							
Major liver resection (three or more Couinaud segments)	27	18 (66.7%)	0 (0%)	9 (33.3%)	1 (1–7)	8 (4–22)	1 (3.7%)
Minor liver resection (less than three Couinaud segments)	25	19 (76%)	2 (8%)	4 (16%)	1 (1–3)	5 (4–13)	0 (0%)
De-roofing giant liver cyst	1	1 (100%)	0 (0%)	0 (0%)	1	5	0 (0%)
Unresectable liver—open/close	2	2 (100%)	0 (0%)	0 (0%)	1 (1–1)	5 (3–7)	2 (100%)
Whipple procedure	32	25 (78.1%)	0 (0%)	7 (21.9%)	3 (1–144)	14 (7–231)	0 (0%)
Distal pancreatectomy	14	3 (21.4%)	1 (7.1%)	10 (71.4%)	1 (1–6)	7 (4–14)	0 (0%)
Total pancreatectomy	1	1 (100%)	0 (0%)	0 (0%)	3	13	0 (0%)
Other pancreatectomy	3	1 (33.3%)	1 (33.3%)	1 (33.3%)	2 (1–5)	18 (3–30)	0 (0%)
Unresectable pancreas—bypass procedure/other	10	8 (80%)	0 (0%)	2 (20%)	1.5 (1–4)	9 (7–95)	1 (10%)
Radical cholecystectomy ± gallbladder fossa resection	10	5 (50%)	3 (30%)	2 (20%)	1 (0–2)	3 (1–10)	0 (0%)
Splenectomy	1	1 (100%)	0 (0%)	0 (0%)	2	5	0 (0%)
Adrenalectomy	2	0 (0%)	0 (0%)	2 (100%)	1 (1–1)	2 (1–3)	0 (0%)
Small bowel resection	8	7 (87.5%)	1 (12.5%)	0 (0%)	1 (0–15)	7.5 (4–15)	1 (12.5%)
Unresectable small bowel—bypass procedure	2	2 (100%)	0 (0%)	0 (0%)	1 (1–1)	5.5 (5–6)	0 (0%)
Completed resections	213	112 (52.6%)	56 (26.3%)	45 (21.1%)	–	–	4 (1.9%)
Total procedures	231	125 (54.1%)	59 (25.5%)	47 (20.3%)	2 (0–144)	10 (1–231)	8 (3.5%)

**Table 3:** Surgical complication grading and frequency

Complication grading	Number (%)
Clavien–Dindo grade I–II	43 (18.6%)
Clavien–Dindo grade III	21 (9.1%)
Clavien–Dindo grade IV	10 (4.3%)
Clavien–Dindo grade V	4 (1.7%)

### Oesophageal Surgery

**Figure 4:**
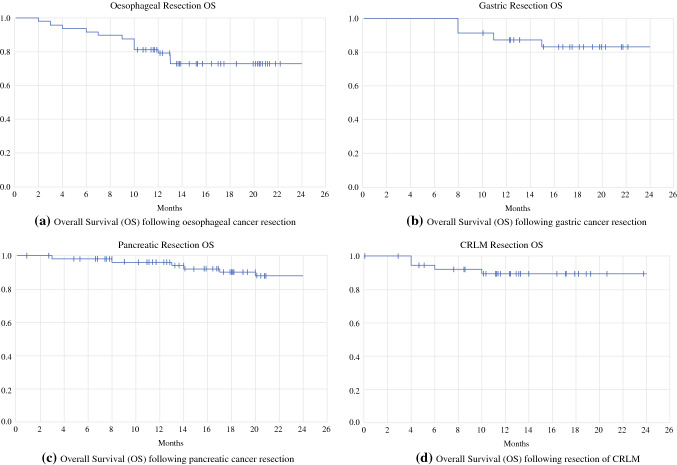
**a**–**d** Kaplan-Meier curves presenting Overall Survival (OS) following **a** gastric cancer resection, **b** oesophageal cancer resection, **c** pancreatic cancer resection and **d** CRLM resection

A total of 48 oesophaegal resections were performed consisting of Ivor–Lewis oesophagectomy (ILO) (*n* = 45), oesophago-gastrectomy (*n* = 2) and oesophago-gastrectomy with right lower lobectomy (*n* = 1). Primary operative approaches were open (*n* = 5), laparoscopic (*n* = 39) and robotic (*n* = 4). Three (6.7%) patients had circumferential margin (CRM) involvement on pathological assessment, two of which were performed as salvage procedures for recurrent squamous cell carcinoma following definitive chemoradiotherapy. Three patients received prolonged neo-adjuvant chemotherapy; all were deemed to have complete response and did not receive post-operative treatment. Thirty-two (88.9%) of patients on a peri-operative treatment pathway went on to receive adjuvant chemotherapy. Two (4.2%) patients died in the peri-operative period, and two (4.2%) patients experienced major morbidity. One patient required reintubation secondary to acute respiratory distress syndrome following ILO and died 2 months post-operatively. One patient required a slow respiratory wean and surgical tracheostomy following ILO, before deteriorating with aspiration pneumonia and dying as a result of declining further intubation. One patient sustained vocal cord paralysis following ILO requiring radiesse vocal cord injection, and one patient required interventional radiology (IR)-guided embolisation for a splenic artery bleeding following ILO. For patients who underwent oesophageal resection during this period, 2-year disease-free survival (DFS) was 70.8% and overall survival (OS) 72.9% (Fig. 4A).

### Gastric Surgery

A total of 39 gastric resections were performed consisting of total gastrectomy (*n* = 22), subtotal gastrectomy (*n* = 2) and gastric or small bowel GIST resections (*n* = 15). Primary operative approaches were open (*n* = 24), laparoscopic (*n* = 9) and robotic (*n* = 6). One patient underwent gastrojejunostomy bypass in view of advanced disease at the time of planned resection; three other bypass procedures were performed for gastric outlet obstruction. Case prioritisation meant that GIST resections were initially deferred, with only one patient undergoing surgery before August 2020. All gastric resections had clear microscopic margins on histopathological assessment. Two patients received prolonged neo-adjuvant chemotherapy with complete response; 15 (88.2%) patients on a peri-operative pathway received adjuvant chemotherapy. There was no mortality following gastric resection, and one patient (2.6%) experienced major morbidity requiring re-intubation on day 1 following total gastrectomy; however, they were successfully extubated 4 days later and discharged home on post-operative day 14. For patients who underwent gastric cancer resection, 2-year disease-free survival (DFS) was 66.7% and overall survival (OS) 83.3% (Fig. 4B).

### Pancreatic Surgery

A total of 50 pancreatic resections were performed consisting of Whipple procedures (*n* = 32), distal pancreatic resections (*n* = 14), total pancreatectomy (*n* = 1) and other pancreatic resections (*n* = 3). Primary operative approaches were open (*n* = 30), laparoscopic (*n* = 2) and robotic (*n* = 18). Ten attempted Whipple’s were abandoned owing to advanced disease, with nine bypass procedures performed. The bypass rate for pancreatic resections was 18%, which is higher than the pre-pandemic 2019 national average, but in keeping with the trend nationwide.^2^ Five (10%) pancreatic resections were deemed R1 resections on histopathological assessment (microscopic resection margin involvement), all involving the superior mesenteric vein (SMV) median margin. Seven patients received upfront chemotherapy, and 22 patients were considered for adjuvant therapy, with 19 (86.4%) receiving chemotherapy or chemoradiotherapy post-operatively. One patient declined adjuvant chemotherapy, and two were deemed unfit for following oncology assessment; two further patients were not assessed following protracted post-operative stay. One patient (2.0%) died following major pancreatic resection, and four (8.0%) experienced major morbidity. One patient required a return to theatre on day 2 for bleeding at the gastrojejunostomy site following a Whipple procedure. They required multiple further returns to theatre, developed persistent abdominal sepsis and died in hospital 6 months later. One patient required a return to theatre and right hemicolectomy for colonic ischaemia following Whipple procedure with partial SMV resection and reconstruction. One patient required re-admission with a delayed post-operative bleed following laparoscopic segmental pancreatectomy requiring IR embolisation of left gastric artery branches. One patient sustained an ischaemic stroke on day 3 following a Whipple procedure; one patient required reintubation with chest sepsis following Whipple resection. For patients who underwent pancreatic cancer resection, 2-year disease-free survival (DFS) was 68.0% and overall survival (OS) 88.0% (Fig. 4C).

### Liver Surgery

A total of 52 livers underwent surgery, consisting of major liver resections (defined as three or more Couinaud segments) (*n* = 27) and minor liver resections (less than three Couinaud segments) (*n* = 25). Primary operative approaches were open (*n* = 37), laparoscopic (*n* = 2) and robotic (*n* = 13). These were for colorectal liver metastases (CRLM) (*n* = 37, 71.2%), cholangiocarcinoma (*n* = 4, 7.7%), hepatocellular carcinoma (HCC) (*n* = 3, 5.8%), benign pathology (*n* = 7, 13.5%) and synchronous CRLM and HCC (*n* = 1, 1.9%). There were two abandoned resections because of advanced disease. Forty-four (97.8%) of eligible patients were considered for further post-operative therapy; 24 (53.3%) commenced adjuvant chemotherapy, 18 (40.0%) were deemed for surveillance and 2 (4.4%) declined chemotherapy. One patient (1.9%) died following liver surgery, and two (3.8%) suffered major morbidity. One patient underwent a left hepatectomy, partial caudate lobectomy with portal vein resection and reconstruction for undifferentiated tumour at the hepatic hilum after systemic chemotherapy following a failed attempt to resect this previously, who developed fulminant liver failure post-operatively with portal vein graft thrombosis and died on post-operative day 3. One patient underwent segmental resection of two CRLMs, developed sepsis post-operatively requiring CCU admission for vasopressor support. One patient underwent segmental resection of three CRLMs and was re-admitted with septic shock secondary to a large peri-hepatic collection with ultrasound-guided drainage for post-operative biloma. They were discharged 6 days later with a drain in situ and underwent outpatient ERCP for biliary decompression, with subsequent CT imaging showing resolution of the collection. For patients who underwent liver resection for CRLM, 2-year disease-free survival (DFS) was 71.1% and overall survival (OS) 89.5% (Fig. 4D).

### Other Surgery

Radical cholecystectomy with or without gallbladder fossa resection (*n* = 10), small bowel resections (*n* = 8), adrenalectomy (*n* = 2), splenectomy (*n* = 1), excision of complex mediastinal mass (*n* = 1), excision of diaphragmatic tumour (*n* = 1) and de-roofing of giant liver cyst (*n* = 1) were performed. One patient died following D3/jejunal resection for a neuroendocrine tumour and developed multi-organ failure post-operatively, on a background of known cold-agglutinin haemolytic anaemia.^13^

### Peri-operative COVID-19

During the study period, one patient (0.43%) contracted COVID-19 peri-operatively following a central liver resection who developed acute dyspnoea on post-operative day 10 and subsequently tested PCR positive for SARS CoV-2. The patient was discharged home on day 16 and made a full recovery, reporting no lasting sequelae at 6-week outpatient clinic review.

## Discussion

The COVID-19 pandemic caused unprecedented challenges and disruption to global healthcare delivery.^5^ At the time of RM Partners Cancer Hub’s conception, London was the epicentre of the pandemic within the UK accounting for 45% of national COVID-19-related mortality, with elective surgery largely halted to maximise critical care capacity.^1^ As the surgical management of UGI malignancy is time critical, developing alternative strategies to continue to provide surgical care became essential.^2,3^ In stage II or III oesophageal, gastric or pancreatic cancers, a delay of only 3 months to surgery can be associated with a > 17% reduction in long-term survival.^14^ In addition, upstaged cancers often prove more costly to treat in terms of surgery and adjuvant therapy.^14^ Every patient on UGI cancer treatment pathways at the beginning of the pandemic was re-discussed in our Specialist Upper GI Multi-disciplinary meeting with only a small number of patients undergoing extended neoadjuvant treatment. Our experience demonstrates that establishing a surgical oncology hub early in the COVID-19 pandemic allowed for the safe and timely delivery of UGI cancer surgery. A total of 213 UGI resections were performed by several UGI surgical departments during the study period, at a time when most NHS hospitals were only able to undertake emergency surgery owing to the re-allocation of resources and personnel for COVID admissions. In addition, we report favourable survival outcomes and morbidity rates emphasising the high standard of perioperative care that was maintained throughout this period.^15,16^ In particular, patients undergoing oesophagectomy and gastrectomy have favourable 1- and 2-year survival compared with the National Oesophago-Gastric Cancer Audit (NOCGA).^17^

The RM Partners Cancer Hub comprised several key facets: (1) a combined ‘hub and spoke’ model of cooperation between hospitals to pool cancer care; (2) the maintenance of a ‘clean site’ for safe operating; (3) rigorous peri-operative screening protocols; and (4) careful patient selection and case prioritisation. The ‘hub and spoke’ model ensured NHS hospitals could continue to provide as much cancer treatment as possible by pooling resources and maximising operating capacity at the protected ‘hub’. This strategy involved working closely with the independent sector, and a provider was used as an alternative ‘clean site’. The requirement for a COVID-19-free surgical pathway was not unique to the RM Partners Cancer Hub; however, it was an essential component in minimising nosocomial infection to the vulnerable cohort of patients with cancer.^1,4, 18–20^ This was achieved by the strict adherence to infection control policies and evolving best practice guidelines. As the Royal Marsden Hospital is a non-acute NHS Trust focussed on cancer treatment without a general emergency department, maintenance of a cold site for operating was feasible. We reported only a single case of peri-operative COVID-19 infection despite a rapid rise of cases in the wider population. This also highlights the success of our peri-operative patient screening strategy and in-hospital pathways at minimising nosocomial infection and enabling the ongoing provision of safe surgical care. This success also stems from the integrated approach and multi-disciplinary expertise of a specialist cancer hospital that regularly undertakes complex surgical interventions. The experience and oncology focus shared by our anaesthetists, intensivists, nursing teams, dieticians, physiotherapists and all healthcare professionals allowed our organisation to scale our services across multiple tumour groups whilst maintaining patient safety and achieving favourable clinical outcomes. Through running ‘cold’ hospital sites with elective ITU access, delays/cancellations are minimised for patients requiring ‘time-sensitive’ cancer surgery; as there are no emergency admissions, elective bed spaces can be protected and last-minute cancellations are reduced.^21^ The establishment of the 31-day decision to treat standard^22^ will place more pressure on acute NHS Trusts, and further consideration may need to be given to increasing access to regional ‘cold cancer treatment sites’ in times of need.

The Hub strategy allowed us to provide the advantages of minimally invasive surgery (MIS) in 46% of operative cases, with no associated peri-operative risk to patients or theatre personnel, once data had shown minimally invasive surgery to be safe. Our UGI case prioritisation schedule was carefully designed to ensure the continuation of surgery for those at highest risk of disease progression, while accounting for associated peri-procedural morbidity. The prioritisation schedule required effective administration and inter-professional cooperation to maintain the equitable delivery of cancer surgery to all patients across the West London Cancer Alliance. It ensured that the most urgent cases were performed in a timely manner and resources were distributed fairly during this time.

The rapid development of a new paradigm for care required in the context of COVID-19 required all healthcare institutions to be flexible and adapt quickly to changes in pathogen transmission. The ‘RM Partners Cancer Hub’ approach provided a workable model for providing multi-disciplinary UGI cancer care and surgery with favourable 2-year DFS and OS compared with nationally published pre- and post-pandemic data. It also established a template for complex oncological surgery during periods of marked disruption to healthcare service delivery and should be a useful guide in the future planning of safe operating pathways.

## Conclusions

The safe provision of surgical care for UGI malignancy is essential for long-term patient survival. Our experience during the COVID-19 pandemic demonstrates that the ‘RM Partners Cancer Hub’ approach, with ’clean site’ operating, appropriate case prioritisation and protocols to minimise peri-operative transmission not only was safe but also allows achievement of favourable clinical outcomes for UGI surgery and should be considered as a standard of care during future periods of healthcare service disruption by a pandemic.
